# Clonal hematopoiesis driven by *Dnmt3a* mutations promotes metabolic disease development in mice

**DOI:** 10.1172/JCI197100

**Published:** 2025-09-30

**Authors:** Bowen Yan, Qingchen Yuan, Marco M. Buttigieg, Prabhjot Kaur, Annalisse R. McKee, Daniil E. Shabashvili, Caitlyn Vlasschaert, Alexander G. Bick, Michael J. Rauh, Olga A. Guryanova

**Affiliations:** 1 University of Florida College of Medicine, Gainesville, Florida, USA.; 2 University of Florida Health Cancer Center, Gainesville, Florida, USA.; 3 Queen’s University, Kingston, Ontario, Canada.; 4 Vanderbilt University Medical Center, Nashville, Tennessee, USA.

**Keywords:** Hematology, Inflammation, Metabolism, Diabetes, Mouse models, Obesity

## Abstract

#ClonalHematopoiesis, an #aging related condition caused by mutations in the #blood system, promotes #obesity, and with high-fat #diet drives the risk of #diabetes and #MASLD

**To the Editor:** Clonal hematopoiesis (CH) is associated with an increased risk of nonhematologic chronic diseases, including metabolic disorders, yet causality remains poorly defined. DNA methyltransferase 3A (*DNMT3A*) is the most altered gene in CH, commonly through monoallelic loss-of-function (LOF) and Arg882His (RH) mutations. Here, we demonstrate in a mouse model that CH driven by *Dnmt3a* RH and especially LOF promotes obesity, diabetes, and chronic liver disease, effects that are further exacerbated by high-fat diet (HFD).

CH is defined as expansion of a blood cell clone marked with somatic mutations absent diagnosis of hematologic malignancies. In addition to a risk of future leukemia, CH is notably associated with chronic nonhematologic diseases, such as inflammatory disorders and cardiovascular disease (1). Epidemiological studies found that CH, especially when driven by *TET2* loss, was enriched in individuals who were overweight and those with type 2 diabetes (T2D) and chronic liver disease (2, 3). Whether CH is a cause or a consequence of these comorbidities — a question of high translational significance — is incompletely understood.

*DNMT3A* is the most mutated gene in CH (4). While the RH variant is enriched in acute myeloid leukemia, most CH-related *DNMT3A* alterations are LOF (5), indicating they may not be fully mechanistically equivalent. The specific impact of CH driven by *DNMT3A*(LOF) versus *DNMT3A*(RH) on chronic disease development has not been investigated.

In meta-analysis across nonobese individuals in the UK Biobank and All of Us cohorts, we found that CH, including *DNMT3A* CH, was associated with incident obesity risk (*DNMT3A* CH HR = 1.14 [95% CI, 1.01–1.27], Figure 1A). To elucidate the functional impact of CH with *DNMT3A* LOF and RH mutations on metabolic disease development, we created a mouse bone marrow transplantation–based chimeric model with 20% of *Dnmt3a^+/–^* (representing LOF), *Dnmt3a^+/R878H^* (corresponding to human RH), or *WT* control cells (marked by pan-leukocytic CD45.2) mixed with 80% *WT* support (CD45.1) to mimic a clinically meaningful large CH clone (Figure 1B and Supplemental Figure 1, A and B; supplemental material available online with this article; https://doi.org/10.1172/JCI197100DS1). Eight weeks after transplantation, to allow hematopoietic reconstitution, and after confirming engraftment, mice were randomized to high-fat high-glucose Western diet (HFD) or normal chow groups. Animals on control chow harboring *Dnmt3a^+/–^* hematopoietic cells exhibited faster body weight gain (1.7-fold, *P* < 0.001) and a moderately higher food intake compared with *Dnmt3a^WT^*-engrafted controls (Figure 1, C and D, and Supplemental Figure 1, C–E), accompanied by subcutaneous white adipocyte hypertrophy (Supplemental Figure 1F and Figure 1E). HFD accentuated bodyweight increase and adipocyte hypertrophy, particularly in the *Dnmt3a^+/–^*-CH group (Figure 1, C and E, and Supplemental Figure 1, D and F), which became overweight and obese earlier than *Dnmt3a^WT^* controls (Supplemental Figure 1E). Furthermore, *Dnmt3a^+/–^*-CH animals exhibited higher plasma leptin (*P* = 0.011) and resistin levels (*P* = 0.048) after 6 weeks of HFD, consistent with increased adiposity (Figure 1F and Supplemental Figure 1G). Even without HFD challenge, plasma leptin was mildly elevated in both *Dnmt3a^+/–^* and *Dnmt3a^+/RH^*-CH animals 6 months after transplantation (corresponding to 4 months after diet randomization) (Supplemental Figure 1, H and I), suggesting a direct obesogenic effect of *Dnmt3a* CH.

In line with a well-established link between overweight, insulin resistance, and diabetes, when maintained on HFD these mice exhibited a greater propensity toward impaired glucose metabolism, with elevated fasting blood glucose and insulin levels and impaired glucose tolerance (Figure 1, G–I, and Supplemental Figure 1J). On normal chow, almost half of *Dnmt3a^+/–^*-CH mice had their fasting glucose reach prediabetic levels 7 months after transplantation despite unperturbed plasma insulin and glucose tolerance (Supplemental Figure 1, K–M). These findings suggest that *Dnmt3a* CH, particularly that driven by *Dnmt3a* LOF, directly contributes to metabolic dysfunction similar to obesity and T2D.

Given the emerging link between CH, inflammation, and changes in myelopoiesis (6), next we examined lineage composition in the hematopoietic system and cytokine profiles. *Dnmt3a^+/–^*-CH mice showed increased abundance of donor-derived (CD45.2) inflammatory monocytes in spleens and peripheral blood compared with both wild-type competitor cells (CD45.1) within the same animal and with *Dnmt3a^WT^*-CH (CD45.2) controls, a finding that was most pronounced under HFD; the *Dnmt3a^+/RH^*-CH group exhibited an intermediate phenotype (Figure 1J and Supplemental Figure 2, A and B). Consistently, after 10 weeks of HFD, *Dnmt3a^+/–^*-CH mice had elevated inflammation-related cytokines (MIP-1α, CXCL1, IL-1α, and IL-15) compared with *Dnmt3a^WT^*-engrafted controls, although levels of classical proinflammatory IL-1β and IL-6 were less perturbed (Figure 1K and Supplemental Figure 2C).

The meta-inflammatory state was further evident in the livers of *Dnmt3a^+/–^*- and *Dnmt3a^+/RH^*-CH animals. On normal chow, both *Dnmt3a*-CH groups exhibited greater lobular hepatic inflammation, with prominent immune infiltrate, compared with *Dnmt3a^WT^* animals (Figure 1L and Supplemental Figure 2, D and E). Furthermore, *Dnmt3a^+/–^*-CH animals developed signs of metabolic dysfunction–associated steatotic liver disease (MASLD) characterized by larger and more numerous macrovesicular fat droplets (Figure 1L and Supplemental Figure 2F). Under HFD, the *Dnmt3a^+/–^*-CH group progressed to severe MASLD, with marked hepatitis reflected by high nonalcoholic fatty liver disease activity score (3) and extensive fibrosis (Figure 1, L–N).

Collectively, our epidemiological and experimental findings indicate that *Dnmt3a*-CH promotes the development of obesity and metabolic disease. In mice, these phenotypes are exacerbated by HFD, most strongly in the *Dnmt3a^+/–^* context. While previous studies focused on inflammation and obesity driving expansion of the CH clone, our findings indicate this relationship is bidirectional, wherein CH directly contributes to obesity and metabolic syndrome, suggesting a vicious molecular etiology cycle. Our study highlights the differential impact of various CH mutations, urging detailed mutation- and gene-specific investigation of CH in human chronic disease pathogenesis, risk stratification, and mitigation through pharmacologic, lifestyle, and dietary interventions.

For detailed methods, information regarding sex as a biological variable, statistics, study approval, author contributions, and acknowledgments, see the Supplemental Methods.

## Funding support

This work is the result of NIH funding, in whole or in part, and is subject to the NIH Public Access Policy. Through acceptance of this federal funding, the NIH has been given a right to make the work publicly available in PubMed Central.

- NIH award R01DK121831 to OAG.
- Edward P. Evans Foundation to OAG.
- Oxnard Family Foundation to OAG.
- Ocala Royal Dames for Cancer Research Inc. to BY.
- ACS Institutional Research Grant to BY through the University of Florida Health Cancer Center (UFHCC).
- Canadian Institutes of Health Research (CIHR) (202010PJT-451137) to MJR.
- Ontario Institute for Cancer Research (CPTRG-056) to MJR.
- New Frontiers in Research Fund (NFRFE-2019-01575) to MJR.
- CIHR Vanier Canada Graduate Scholarship to MMB.
- Sinclair Graduate Scholarship in Cancer Research to MMB.
- NIH grant P30CA247796 to UFHCC, a National Cancer Institute–designated cancer center.

## Supplementary Material

Supplemental data

Unedited blot and gel images

Supporting data values

## Figures and Tables

**Figure 1:**
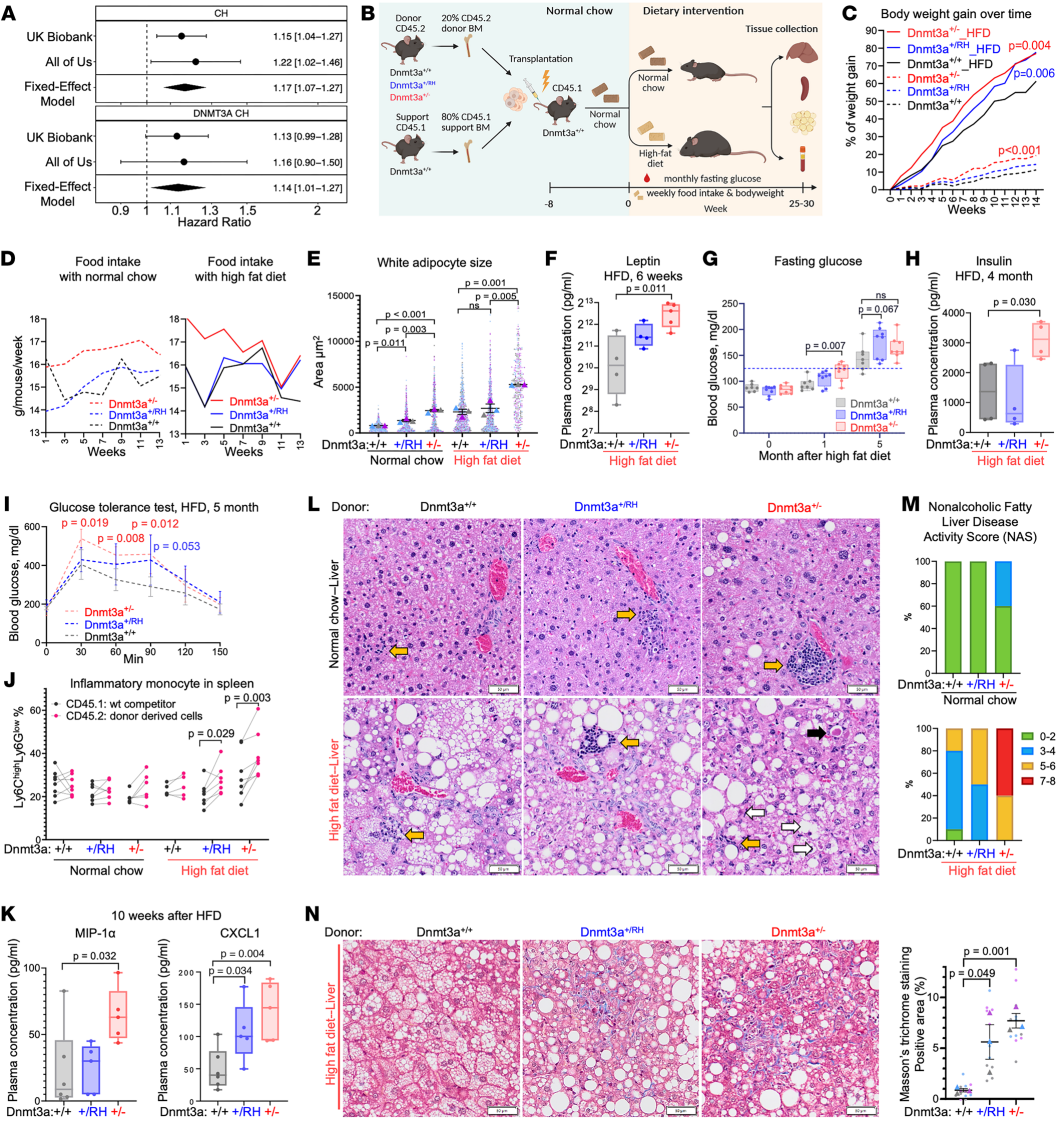
Experimental *Dnmt3a* CH promotes obesity, impaired glucose metabolism, inflammation, and severe steatohepatitis. (**A**) CH and incident obesity risk; Cox proportional hazard regression. (**B**) Experimental workflow. (**C**) Body weight gain; mixed-effects analysis vs. *Dnmt3a^+/+^* control; *n* = 7–8. (**D**) Food intake, averaged per cage. (**E**) Adipocyte size (SuperPlot, *n* = 3/group, 2 fields/animal); unpaired 2-tailed *t* test. (**F**–**H**) Plasma leptin (**F**), fasting glucose (**G**), and insulin (**H**) levels on HFD, plotted as quartiles. (**I**) Blood glucose levels after glucose load in fasted animals; mean ± SEM; 2-tailed Student’s *t* test vs. *Dnmt3a^+/+^* controls. (**J**) Inflammatory monocytes in donor (CD45.2) or wild-type competitor/host (CD45.1) myeloid cells in spleen; paired 2-tailed Student’s *t* test. (**K**) Plasma MIP-1α and CXCL1; unpaired 2-tailed *t* test. (**L**) Liver histopathology (H&E; black arrow, acidophilic body; white arrows, ballooned hepatocytes; yellow arrows, inflammatory infiltrates; scale bar: 50 μm). (**M**) Steatohepatitis severity score (*n* = 3/group, 5 fields/animal). (**N**) Liver fibrosis (Masson’s trichrome staining) and percentage of positive area; unpaired 2-tailed *t* test with Welch’s correction (SuperPlot, *n* = 3/group, 4 fields/animal).

## Version 1 09/30/2025

In-Press Preview

## Version 2 12/01/2025

Electronic publication
