# Supplementary material for: Clonal hematopoiesis driven by Dnmt3a mutations promotes metabolic disease development in mice
Source: J Clin Invest. 2025 Sep 30;135(23):e197100. doi: 10.1172/JCI197100 (PMC12646656; doi:10.1172/JCI197100)
Supplement: Supplemental data [file jci-135-197100-s300.pdf]

Clonal hematopoiesis driven by *Dnmt3a* mutations promotes metabolic disease development in mice

Bowen Yan, Qingchen Yuan, Marco M. Buttigieg, Prabhjot Kaur, Annalisse R. Mckee, Daniil E. Shabashvili, Caitlyn Vlasschaert, Alexander G. Bick, Michael J. Rauh, Olga A. Guryanova

SUPPLEMENTARY INFORMATION

Supplementary Figure 1

Hematopoietic-specific *Dnmt3a* alterations in a model of CH promote obesity and impaired glucose tolerance

Supplementary Figure 2

Model CH with *Dnmt3a* deficiency or mutation promotes inflammation, advanced steatohepatitis, and liver damage

Supplementary Methods

Author Contributions

Acknowledgements

Supplementary References

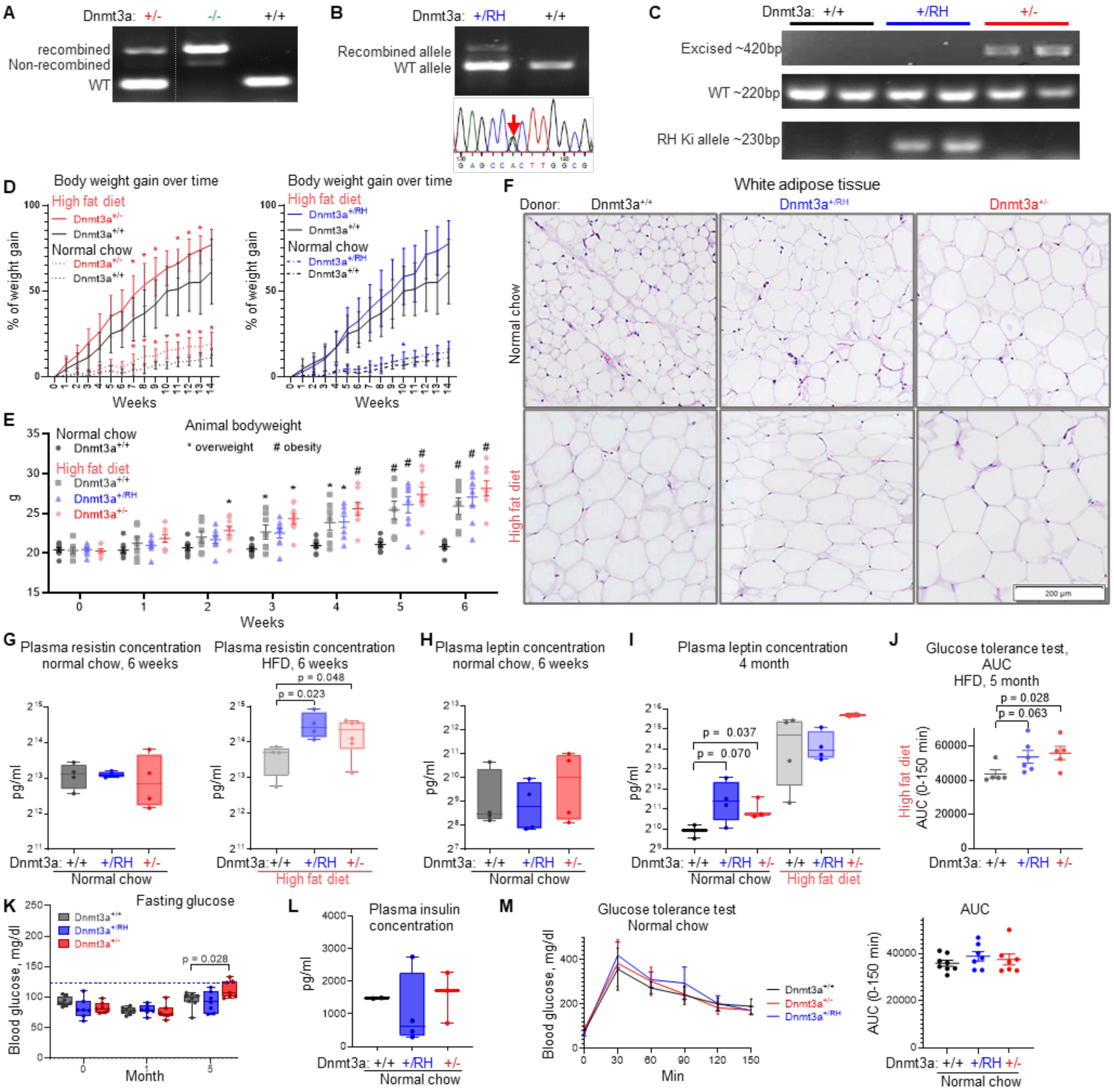

Supplementary Figure 1. Hematopoietic-specific *Dnmt3a* alterations in a model of CH promote obesity and impaired glucose tolerance

(A-B) Genotyping of donor mice confirms full recombination of the inducible knock-out or knock-in alleles. Complete excision of the *Dnmt3a* knock-out allele was detected by PCR on genomic DNA purified from peripheral blood nucleated cells (A). Recombination allowing expression of the $Dnmt3a^{R878H}$ knock-in allele was detected using genomic DNA PCR followed by Sanger sequencing of peripheral blood nucleated cell cDNA (B). (C) Genotyping of peripheral blood from recipient mice 4 months after bone marrow transplantation (BMT) confirms consistent presence of donor-derived cells. (D) Animal body weight gain over time; mean ± SEM; Student's *t*-test vs $Dnmt3a^{+/+}$ controls under same diet condition, *p < 0.05, n=7-8/group. (E) Animal body weight (n≥7). Mice are considered overweight if their body weight exceeds that of age- and sex-matched controls by 10–20% and obese by more than 20%. (F) White adipose tissue section stained with H&E (bar – 200 µm). (G) Plasma resistin levels in indicated groups, unpaired *t*-test. Plasma samples were collected at 14 weeks post BMT (6 weeks after diet randomization). (H-I) Plasma leptin levels in indicated groups, 6 weeks (H) or 4 months (I) after randomization to HFD or control chow, unpaired *t*-test. (J) Calculated area under the curve (AUC) of glucose

tolerance test after 5 months of HFD; mean ± SEM; Student's *t*-test vs *Dnmt3a*$^{+/+}$ controls. (K) Fasting blood glucose levels at baseline and at 1 or 5 months after diet randomization in a control normal chow group, unpaired *t*-test. (L) Plasma insulin concentration at 4 months after diet randomization in control normal chow group. (M) Glucose tolerance test (GTT) and calculated area under the curve (AUC) in indicated groups under normal chow; mean ± SEM , n≥5 for all groups.

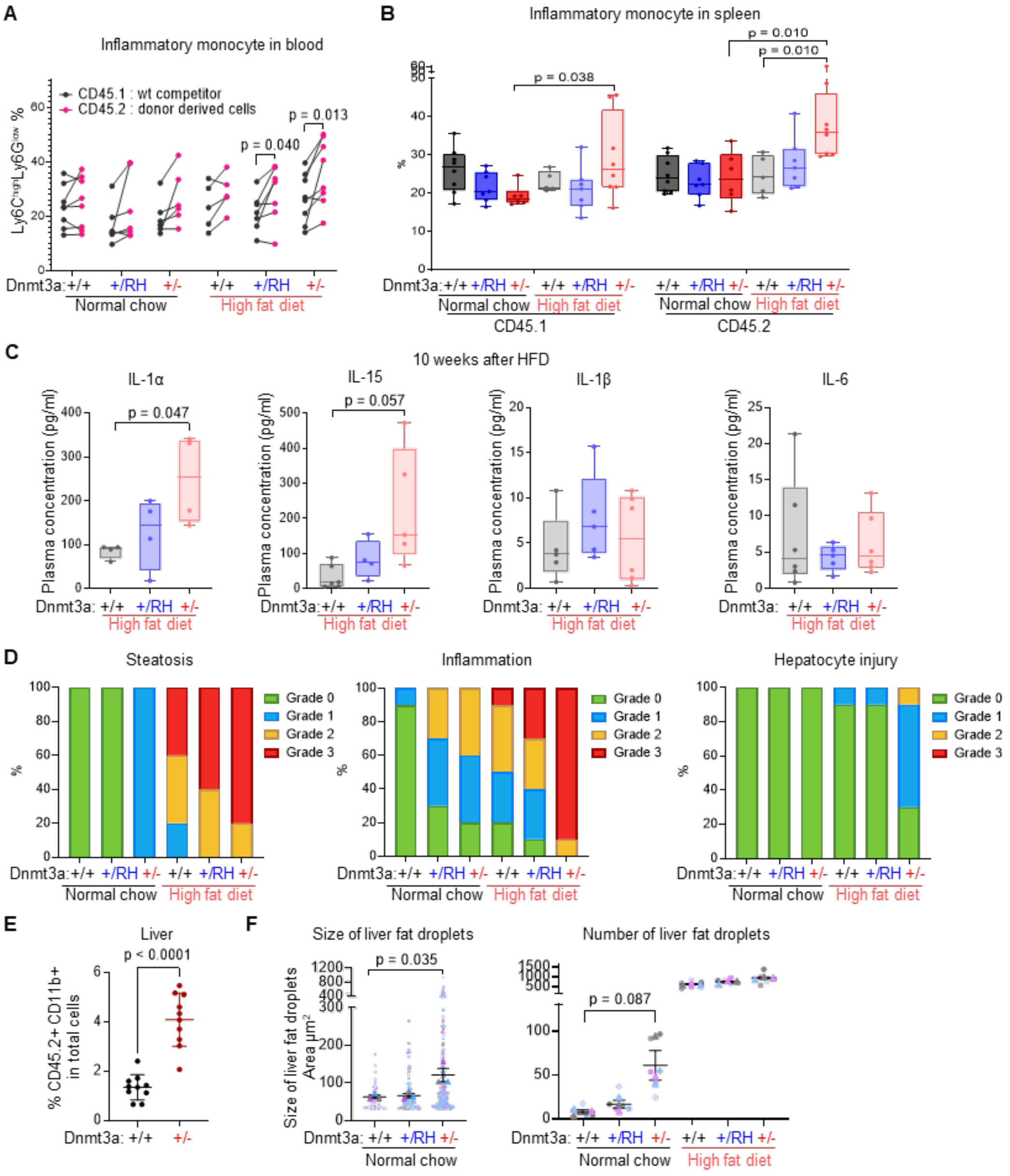

Supplementary Figure 2. Model CH with *Dnmt3a* deficiency or mutation promotes inflammation, advanced steatohepatitis and liver damage

(A-B) Proportion of inflammatory monocytes in CD45.1^{+}CD11b^{+} or CD45.2^{+}CD11b^{+} population in peripheral blood, paired Student's *t*-test (A) and in spleens, unpaired Student's *t*-test with Welch's correction (B). (C) Levels of IL-1α, IL-15, IL-1β and IL-6 in blood plasma, plotted as quartiles, all points are shown; unpaired *t*-test with Welch's correction. (D) Steatohepatitis was graded using nonalcoholic steatohepatitis histologic criteria (n=3 per group, 5 fields per animal). (E) Percentage of CD45.2^{+} myeloid cells in liver, mean ± SEM, Student's *t*-test. (F) Size and number of liver fat droplets with SuperPlot (n=3 per group, 2 fields per animal, unpaired Student's *t*-test, Welch's correction was applied when analyzing the number of liver fat droplets).

Supplementary Methods

Sex as a biological variable

For human cohort studies, sex along with other potential confounding factors were adjusted for in multivariate Cox proportional hazard regression modeling time to incident obesity diagnosis.

For animal models, all recipient mice used for bone marrow transplantation in this study were immune-competent, healthy females. Based on our prior studies, no sex-specific differences were expected or tested for.

Human Cohort Studies

Data from participants in the UK Biobank late 2023 release (*n*=333,808) and All of Us Research Program version 7 release (*n*=126,675) cohorts was used for this study. CH genotype information was derived from peripheral blood whole exome sequencing and whole genome sequencing in the UK Biobank and All of Us, respectively, using previously described methods(1). CH was defined based on the presence or absence of a somatic driver mutation with a variant allele fraction (VAF) ⩾2% as per WHO guidelines(2). *DNMT3A* and non-*DNMT3A* CH driver status was determined based on the somatic driver mutation with the largest VAF in an individual.

Obesity was defined using electronic health record data collected by the UK Biobank (Field Code 130792 – Date E66 First Reported Obesity) and All of Us (Concept ID 433736 – Obesity). Participants were excluded from incident risk analysis if they had a history of obesity or if their body mass index (BMI) at study enrollment was ⩾30. Due to possible weight bias amongst healthcare professionals and the social stigma surrounding obesity(3), we acknowledge that there may be bias in reporting of obesity diagnoses in electronic health records which is a limitation of our study. Time to incident obesity diagnosis was modelled with multivariate Cox proportional hazard regression using survival v3.6-4 adjusted for age, age^2, sex, smoking, genetic principal components 1-10, socioeconomic deprivation index (UK Biobank: Field Code 22189 – Townsend deprivation index at recruitment; All of Us: ZIP Code Socioeconomic Status Data – Deprivation Index), and participant BMI at baseline. Meta-analysis of results from the UK Biobank and All of Us was performed with metafor v4.8-0 using a fixed-effects model. Hazard ratios and 95% confidence intervals were visualized using ggplot2 v3.5.1. All analyses were conducted using R v4.4.0.

Animals

Mice were housed in a specific pathogen-free animal care facility with a controlled housing temperature (23°C) and 12-hour light/12-hour dark cycle. Conditional *Dnmt3a* knockout (*Dnmt3a*$^{+/fl}$, originally generated by the group of Rudolph Jaenisch), *Dnmt3a*$^{+/R878H}$ (corresponds to the *DNMT3A* R882H mutation in humans, Jackson Laboratory stock # 031514) knock-in, and *Dnmt3a*$^{+/+}$ mouse lines with *Mx1-Cre* deleter (JAX stock # 003556) on a C57BL/6J background (JAX stock # 000664) were generated via in-house breeding(4-6). To achieve inducible hematopoietic-specific excision, 8- to 12-week-old *Dnmt3a*$^{+/R878H}$:*Mx1-Cre* and *Dnmt3a*$^{+/fl}$:*Mx1-Cre* mice received five intraperitoneal injections of poly(I:C) (InvivoGen, 20mg/kg of body weight). Poly(I:C)-treated *Dnmt3a*$^{+/+}$:*Mx1-Cre* mice are used as controls. *Mx1-Cre*–driven recombination was validated by PCR using genomic DNA purified from peripheral blood mononuclear cells two weeks after the final

injection. Successful expression of the *Dnmt3a*^{R878H} point mutant is further verified by Sanger sequencing of the peripheral blood mononuclear cell cDNA. All recipient mice used for bone marrow transplantation in this study were C57BL/6.SJL (CD45.1, JAX stock # 002014) immune-competent, healthy females procured from the Jackson Laboratory. Animals had *ad libitum* access to water and either high-fat (42 kcal% fat, TD.88137, Envigo) or standard chow (Teklad 2918 18% protein, Envigo) rodent diet.

Bone marrow transplantation (BMT)

Bone marrow (BM) cells were isolated from C57BL/6 (CD45.2) age-matched, fully recombined *Dnmt3a*$^{+/fl}$, *Dnmt3a*$^{+/R878H}$, and *Dnmt3a*$^{+/+}$ control donors, as well as from congenic C57BL/6.SJL (CD45.1) mice as wild-type support BM. As indicated in Figure 1B, lethally irradiated (5.4 Gy, twice) 6-week-old CD45.1 recipient mice received 20% individual genotype donor BM cells (CD45.2) and 80% wild-type CD45.1 BM cells through a tail vein injection (2×10^5 CD45.2$^+$ donor BM cells and 8×10^5 wild-type CD45.1$^+$ BM cells with a total of 1×10^6 BM cells/recipient mouse). Animals were maintained on a normal chow diet for 8 weeks to allow full engraftment. After hematopoietic reconstitution was confirmed by peripheral blood flow cytometry analysis for CD45.1/CD45.2 chimerism, major mature lineages, and by CBCs at 8 weeks post-BMT, animals were randomly assigned to high fat diet or remained on normal chow.

Flow cytometry analysis

The blood and spleen single-cell suspensions were lysed with an RBC lysis buffer and then resuspended in phosphate buffered saline (PBS) containing 0.2% bovine serum albumin (BSA) (Sigma). The cells were then stained with fluorochrome-conjugated antibodies and analyzed by multiparameter flow cytometry performed using LSR Fortessa instrument (BD Biosciences). Data were analyzed using FlowJo software (v10.4.1). The following antibodies were used:

Antibody	Fluorophore	Company	Catalog number	Dilution
CD45.1	PacBlue	BioLegend	110722	1:200
CD45.2	BV605	BioLegend	109841	1:200
B220	PE-Cy7	BioLegend	103222	1:200
CD11b	AF700	BioLegend	101222	1:200
Gr1	APC	BioLegend	108412	1:200
Ly6G	BV785	BioLegend	127645	1:200

Plasma cytokine and metabolic hormone analysis

Plasma was separated from peripheral blood and sent to Eve Technologies (Canada) for mouse cytokine/chemokine and metabolic hormone (array MD44-plex and MRDMET12) analysis. Results are expressed as pg/mL of plasma.

Glucose Tolerance Test

Animals were fasted overnight, and glucose was administered by intraperitoneal injection at a dose of 1.5 g/kg. Blood glucose levels were measured using a blood glucose meter (AgaMatrix) immediately prior to dosing (0 min) and at 15, 30,

60, 90, 120, and 150 minutes post-loading via tail vein bleeding. Data are presented as mean ± standard error. Statistical difference between group means compared to *Dnmt3a*$^{+/+}$ control group at individual time point was assessed by Student's *t*-test; with $p<0.05$ considered significant (n⩾5 animals per group).

Histological analysis by H&E and Masson's trichrome staining

Animals were euthanized by CO_2 asphyxiation, and harvested tissues were immediately fixed in 10% neutral buffered formalin solution (Sigma, MFCD00003274) for 24 hours. The samples were then submitted to the UF Molecular Pathology Core for processing. Tissues were paraffin-embedded, sectioned, and stained following standard protocols. Slides were imaged using Olympus VS200 Slide Scanner. White adipocyte size was quantified by measuring the cell area using "Analyze Particles" function in ImageJ software (v1.53k). Cells touching the image borders were excluded from the analysis to ensure accurate measurements. Steatohepatitis was evaluated according to modified CRN criteria(7, 8), through histological grading criteria as follows:

Histological grading criteria for steatohepatitis in mice

Steatosis	
Grade 0	<5% of hepatocytes
Grade 1	< 33% of hepatocytes
Grade 2	33-66% of hepatocytes
Grade 3	> 66% of hepatocytes
Inflammation	
Grade 0	Absent
Grade 1	<100 inflammatory cells per focus or <3 inflammatory foci per 20× field
Grade 2	100-500 inflammatory cells per focus or 3-4 inflammatory foci per 20× field
Grade 3	>500 inflammatory cells per focus or >4 inflammatory foci per 20× field
Hepatocyte Injury/Ballooning	
Grade 0	Absent
Grade 1	Rare balloon cells
Grade 2	Widespread hepatocyte ballooning and apoptosis
Modified NASH activity score (NAS)	
Grade 1	0-2 points
Grade 2	3-4 points
Grade 3	5-6 points
Grade 4	7-8 points

Liver fibrosis was quantified by measuring area fraction of blue-stained collagen of Masson's trichrome staining with ImageJ software (v1.53k). Statistical analysis was performed using two-tailed unpaired Student's *t*-test.

Statistics

Animal bodyweight gain was analyzed using mixed-effects analysis. Unpaired comparisons between two groups were performed using Student's *t*-test for normally distributed data with equal variances, Welch's correction for unequal variances, or the Mann–Whitney *U*-test or Kolmogorov-Smirnov test for non-normal data or unequal variances, as appropriate. Data normality was tested using the Shapiro-Wilk test. Pairwise comparisons within the same mouse, comparing the inflammatory monocyte percentage in the $CD45.1^{+}CD11b^{+}$ and $CD45.2^{+}CD11b^{+}$ populations in the spleen and peripheral blood, were conducted using paired Student's *t*-test. Area under curve (AUC) was calculated using the trapezoidal rule. The lowest glucose value for each mouse was subtracted from the AUC. All statistical analyses were carried out with GraphPad Prism 10.3.1. SuperPlot were generated as described previously(9), using three distinct colors—grey, purple, and blue—to distinguish measurements from different biological samples. All data points (dots) corresponding to the same animal were plotted in the same color, with lighter or darker shades indicating technical replicates or repeated measurements within each biological sample. The mean value for each biological sample appears as a triangle, matching the color used for its data points. Statistical analyses were conducted using data averaged per biological replicate. All graphs were created in GraphPad Prism version 10.3.1.

Study approval

Access to the UK Biobank dataset was provided under application 101583. Access to All of Us was provided via institutional agreements established by Queen's University. Local approval for secondary analyses of human data from the UK Biobank and All of Us was obtained from the Queen's University institutional review board.

All animal experiments were conducted in accordance with ethical guidelines and were approved by the Institutional Animal Care and Use Committee (IACUC) at the University of Florida (UF).

Data availability

CH calls for UK Biobank participants have been returned to the UK Biobank Access Management System and will be made accessible to other registered researchers once processed. CH calls for All of Us participants will be shared with registered researchers upon request.

All data point values associated with the main manuscript and supplementary graphs are provided in the "Supporting data values.xlsx" file online.

Author contributions

BY designed the study, performed experiments, analyzed data, and wrote the manuscript. MMB and MJR analyzed the UF Biobank and the All of Us data, with support from CV and AGB. QY performed experiments, contributed to data interpretation, and edited the manuscript. PK, ARM, and DES performed experiments. OAG conceived the study, supervised the study, co-wrote the manuscript, and secured funding. All authors revised and approved the final manuscript.

Acknowledgements

We acknowledge the contributions of the UK Biobank and All of Us programs and participants. This research has been conducted using the UK Biobank Resource under Application Number 101583, and under an All of Us Research Program Data Use and Registration Agreement. This work was supported in part by NIH award R01DK121831 to OAG. OAG was also supported by the Edward P. Evans Foundation, the Oxnard Family Foundation. BY is supported by the Ocala Royal Dames for Cancer Research, Inc. and the ACS Institutional Research Grant to the University of Florida Health Cancer Center (UFHCC). UFHCC is an NCI-designated cancer center (P30CA247796). Flow cytometry analyses were performed at the UF Interdisciplinary Center for Biotechnology Research (ICBR) RRID:SCR_019119. MJR acknowledges funding from the CIHR (202010PJT-451137), OICR (CPTRG-056) and NFRF-E (NFRFE-2019-01575) that supported this work. MMB was supported by a CIHR Vanier Canada Graduate Scholarship and Sinclair Graduate Scholarship in Cancer Research.

This work is, in part, the result of NIH funding, and is subject to the NIH Public Access Policy. Through acceptance of this federal funding, the NIH has been given a right to make the work publicly available in PubMed Central.

Supplementary references

1. Vlasschaert C, Mack T, Heimlich JB, Niroula A, Uddin MM, Weinstock J, et al. A practical approach to curate clonal hematopoiesis of indeterminate potential in human genetic data sets. *Blood.* 2023;141(18):2214-23.
2. Khoury JD, Solary E, Abla O, Akkari Y, Alaggio R, Apperley JF, et al. The 5th edition of the World Health Organization Classification of Haematolymphoid Tumours: Myeloid and Histiocytic/Dendritic Neoplasms. *Leukemia.* 2022;36(7):1703-19.
3. Lawrence BJ, Kerr D, Pollard CM, Theophilus M, Alexander E, Haywood D, et al. Weight bias among health care professionals: A systematic review and meta-analysis. *Obesity (Silver Spring).* 2021;29(11):1802-12.
4. Guryanova OA, Shank K, Spitzer B, Luciani L, Koche RP, Garrett-Bakelman FE, et al. DNMT3A mutations promote anthracycline resistance in acute myeloid leukemia via impaired nucleosome remodeling. *Nat Med.* 2016;22(12):1488-95.
5. Venugopal K, Feng Y, Nowialis P, Xu H, Shabashvili DE, Berntsen CM, et al. DNMT3A Harboring Leukemia-Associated Mutations Directs Sensitivity to DNA Damage at Replication Forks. *Clin Cancer Res.* 2022;28(4):756-69.
6. Feng Y, Yuan Q, Newsome RC, Robinson T, Bowman RL, Zuniga AN, et al. Hematopoietic-specific heterozygous loss of Dnmt3a exacerbates colitis-associated colon cancer. *J Exp Med.* 2023;220(11).
7. Chalasani N, Younossi Z, Lavine JE, Charlton M, Cusi K, Rinella M, et al. The diagnosis and management of nonalcoholic fatty liver disease: Practice guidance from the American Association for the Study of Liver Diseases. *Hepatology.* 2018;67(1):328-57.
8. Wong WJ, Emdin C, Bick AG, Zekavat SM, Niroula A, Pirruccello JP, et al. Clonal haematopoiesis and risk of chronic liver disease. *Nature.* 2023;616(7958):747-54.
9. Lord SJ, Velle KB, Mullins RD, and Fritz-Laylin LK. SuperPlots: Communicating reproducibility and variability in cell biology. *J Cell Biol.* 2020;219(6).
