# Supplementary material for: Clonal hematopoiesis driven by Dnmt3a mutations promotes metabolic disease development in mice
Source: J Clin Invest. 2025 Sep 30;135(23):e197100. doi: 10.1172/JCI197100 (PMC12646656; doi:10.1172/JCI197100)
Supplement: Unedited blot and gel images [file jci-135-197100-s301.pdf]

A

Dnmt3a: +/- -/- +/+

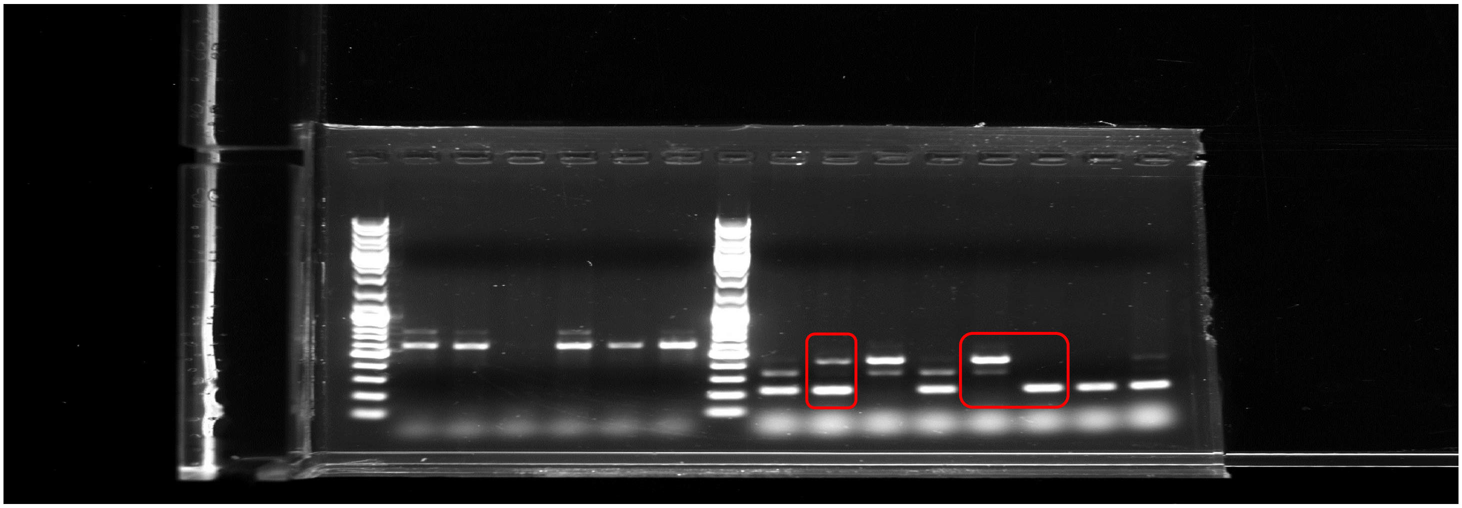

B

Dnmt3a: +/RH +/+

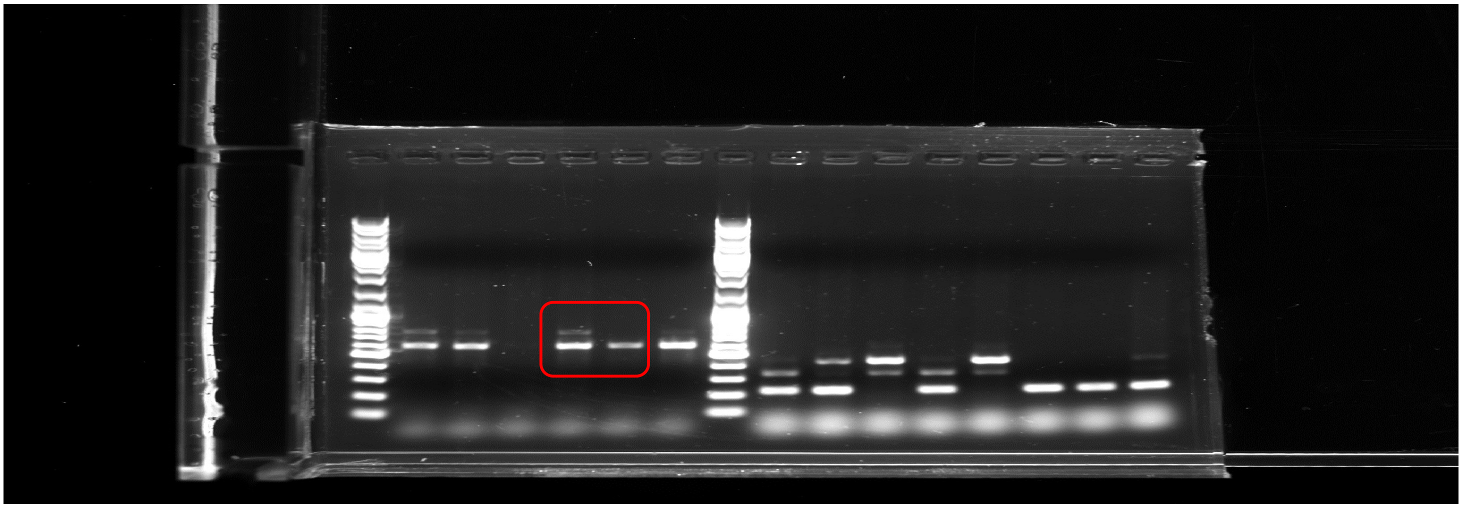

Supplementary Figure 1

C

Dnmt3a: +/+ +/RH +/-

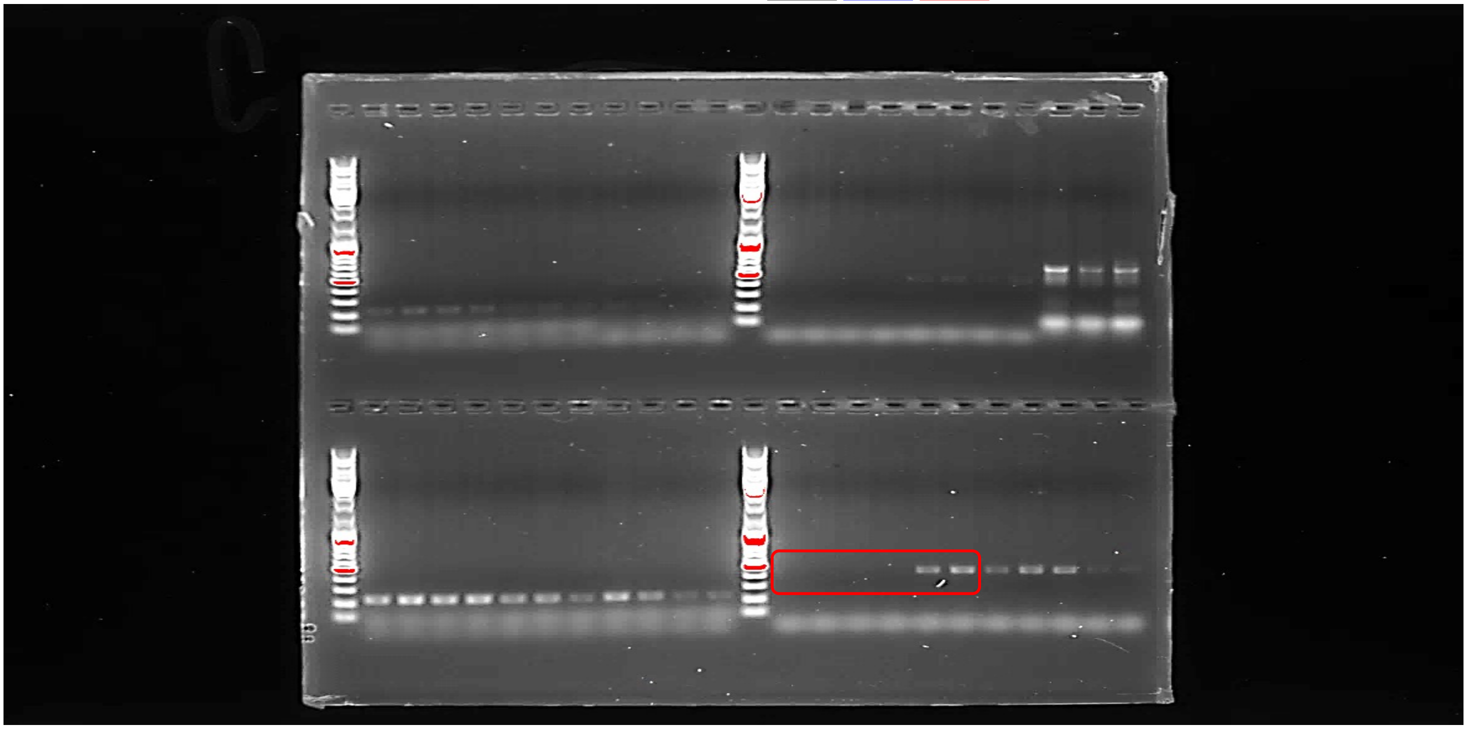

Excised ~420bp

Dnmt3a: +/+ +/RH +/-

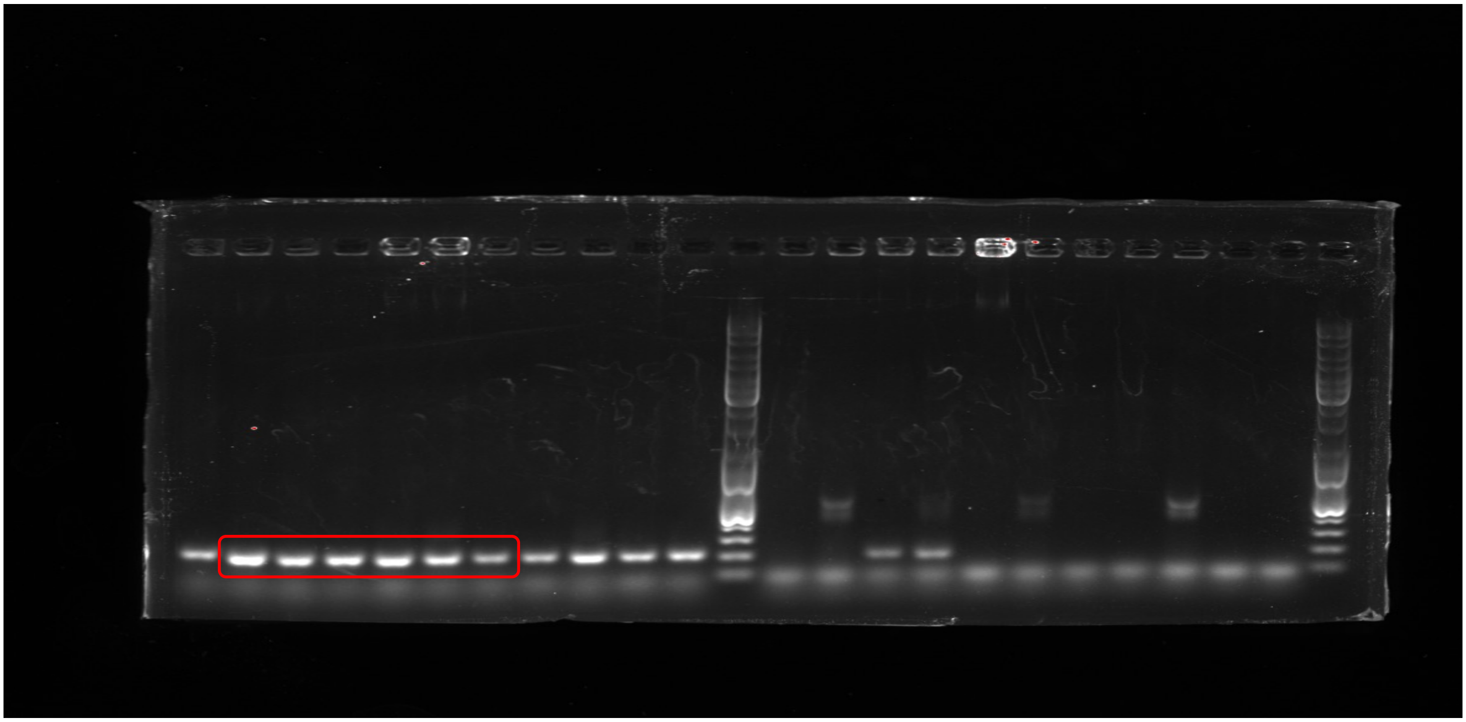

WT ~220bp

Supplementary Figure 1

C

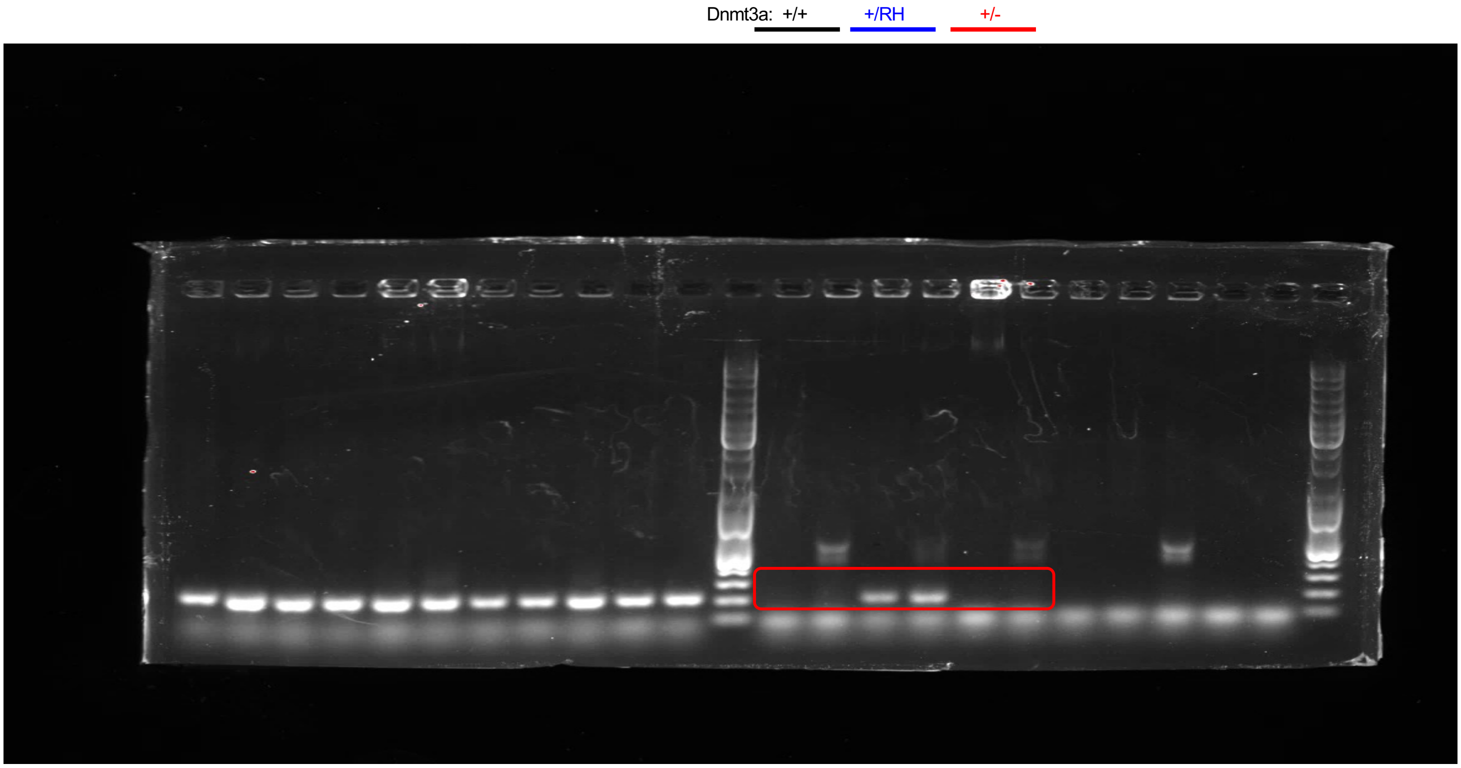

RH Ki allele ~230bp

Supplementary Figure 1
